# Physical activity after cardiac arrest; protocol of a sub-study in the Targeted Hypothermia versus Targeted Normothermia after Out-of-Hospital Cardiac Arrest trial (TTM2)

**DOI:** 10.1016/j.resplu.2021.100076

**Published:** 2021-01-29

**Authors:** Katarina Heimburg, Gisela Lilja, Åsa B Tornberg, Susann Ullén, Erik Blennow Nordström, Hans Friberg, Niklas Nielsen, Lisa Gregersen Østergaard, Anders M. Grejs, Helen Hill, Thomas R. Keeble, Hans Kirkegaard, Marco Mion, Christian Rylander, Magnus Segerström, Johan Undén, Matthew P. Wise, Tobias Cronberg

**Affiliations:** a Lund University, Skane University Hospital, Department of Clinical Sciences Lund, Neurology, Lund, Sweden; b Lund University, Department of Health Sciences, Lund, Sweden; c Skane University Hospital, Clinical Studies Sweden – Forum South, Lund, Sweden; d Lund University, Skane University Hospital, Department of Clinical Sciences Lund, Anesthesiology and Intensive Care, Malmö, Sweden; e Lund University, Helsingborg Hospital, Department of Clinical Sciences Lund, Anesthesiology and Intensive Care, Lund, Sweden; f DEFACTUM, Central Denmark, Region Aarhus, Denmark; g Department of Physiotherapy and Occupational Therapy Aarhus University Hospital, Denmark; h Department of Intensive Care Medicine, Aarhus University Hospital, Aarhus, Denmark; i Adult Critical Care, University Hospital of Wales, Cardiff, United Kingdom; j Essex Cardiothoracic Centre, Basildon and Thurrock University Hospitals, Basildon, United Kingdom; k Department of Allied Health and Medicine, Anglia Ruskin School of Medicine, Chelmsford, United Kingdom; l Aarhus University Hospital and Aarhus University, Emergency Department and Department of Clinical Medicine, Aarhus, Denmark; m Sahlgrenska Academy, University of Gothenburg, Institute of Clinical Sciences, Department of Anesthesiology and Intensive Care Medicine, Gothenburg, Sweden; n Sahlgrenska University Hospital, Department of Neurocare, Unit of Neuropsychology, Gothenburg, Sweden; o Lund University, Skane University Hospital, Department of Clinical Sciences Malmö, Anesthesiology and Intensive Care Medicine, Lund, Sweden; p Operation and Intensive Care, Hallands Hospital Halmstad, Halmstad, Sweden; q Cardiff University School of Medicine, Cardiff, United Kingdom

**Keywords:** Cardiac arrest, Coronary artery disease, Myocardial infarction, Physical activity, Kinesiophobia, Outcome

## Abstract

**Aims:**

The primary aim of this study is to investigate whether out-of-hospital cardiac arrest (OHCA) survivors have lower levels of self-reported physical activity compared to a non-cardiac arrest (CA) control group who had acute myocardial infarction (MI). Additional aims are to explore potential predictors of physical inactivity (older age, female gender, problems with general physical function, global cognition, mental processing speed/attention, anxiety symptoms, depression symptoms, kinesiophobia, fatigue), and to investigate the relationship between self-reported and objectively measured physical activity among OHCA-survivors.

**Methods:**

The Targeted Hypothermia versus Targeted Normothermia after Out-of-Hospital Cardiac Arrest trial (TTM2-trial) collects information regarding age, gender, self-reported physical activity, general physical function, global cognition and mental processing speed/attention at 6 months after OHCA. In this TTM2-trial cross-sectional prospective sub-study, participants at selected sites are invited to an additional follow-up meeting within 4 weeks from the main study follow-up. At this meeting, information regarding anxiety symptoms, depression symptoms, kinesiophobia and fatigue is collected. The OHCA-survivors are then provided with an objective measure of physical activity, a hip-placed accelerometer, to wear for one week, together with a training diary. At the end of the week, participants are asked to once again answer two self-reported questions regarding physical activity for that specific week. MI-controls attend a single follow-up meeting and perform the same assessments as the OHCA-survivors, except from wearing the accelerometer. We aim to include 110 OHCA-survivors and 110 MI-controls in Sweden, Denmark and the United Kingdom.

**Conclusion:**

The results from this sub-study will provide novel information about physical activity among OHCA-survivors.

**Trial registration:**

Registered at ClinicalTrials.gov: NCT03543332, date of registration June 1, 2018

## Highlights

• The results of this study will provide novel information about physical activity among OHCA-survivors.
• Physical activity after a cardiac arrest is currently an important knowledge-gap.
• The results of this study have a potential to improve support and rehabilitation for OHCA-survivors.

## Introduction

Coronary artery disease (CAD) is the most common cause of out-of-hospital cardiac arrest (OHCA) 1, 2. One of the major risk factors for CAD is physical inactivity 3, 4. Physical activity is a preventive strategy for CAD, and physical exercise training is an important part of cardiac rehabilitation 5, 6. International guidelines recommend moderate intensity physical activity for 30 min, 5 days weekly for primary prevention 4, 7 and moderate to vigorous physical activity for at least 20 min, 3–5 times weekly for secondary prevention of CAD 5, 6, 7, 8, 9, 10, 11, 12. Many individuals who had a myocardial infarction (MI) are not physically active to these recommended levels ^13^. Previous studies have identified poor functional capacity ^14^, cognitive impairment ^15^, depression ^16^ kinesiophobia (fear of movement) ^17^ and fatigue ^18^ as factors negatively affecting the level of physical activity and adherence to exercise-based rehabilitation among CAD-patients ^19^. OHCA-survivors often experience additional physical, cognitive and emotional problems related to brain injury secondary to the circulatory arrest 20, 21, 22, 23, 24. They may therefore have higher risk of physical inactivity, compared to other populations of CAD-patients, for whom current exercise-based cardiac rehabilitation programs have been designed. In a previous study, OHCA-survivors with cognitive impairment had lower exercise capacity ^25^. Another study on self-reported cardiac-anxiety among CA-survivors showed that 11–15% avoid physical activities due to concerns about their heart ^26^. Other factors related to physical activity are age and gender 24, 27.

The Targeted Hypothermia versus Targeted Normothermia after Out-of-Hospital Cardiac Arrest trial (TTM2-trial, clinicaltrials.gov NCT02908308) is an international multi-center study comparing targeted temperature of 33℃ post-arrest to targeted normothermia with early treatment of fever ^28^. This study is a cross-sectional prospective sub-study within the TTM2-trial focusing on physical activity.

The primary aim of this study is to investigate whether OHCA-survivors have lower levels of self-reported physical activity compared to a non-CA control group who had acute MI. Additional aims are to explore potential predictors of physical inactivity (older age, female gender, problems with general physical function, global cognition, mental processing speed/attention, anxiety symptoms, depression symptoms, kinesiophobia and fatigue), and to investigate the relationship between self-reported and objectively measured physical activity among OHCA-survivors.

Our hypotheses are:

1 OHCA-survivors will have lower levels of self-reported physical activity compared to MI-controls.
2 Older age, female gender, problems with general physical function, global cognition and mental processing speed/attention, anxiety symptoms, depression symptoms, kinesiophobia and fatigue will be predictors of lower levels of self-reported physical activity amongst OHCA-survivors.
3 Self-reported levels of physical activity among OHCA-survivors will show at least moderate agreement with objectively measured levels of physical activity.

## Methods

### Study population

The TTM2 main trial has included 1900 adult OHCA-survivors of a presumed cardiac origin ^28^. This sub-study is performed at 8 of the 61 TTM2 sites in Sweden (n = 5), Denmark (n = 1) and the United Kingdom (n = 2). Inclusion and exclusion criteria for the TTM2 trial have been described ^28^. Additional exclusion criteria for this sub-study are >80 years of age, a Clinical Frailty Score ^29^ of 8 or 9 before the CA, a dementia diagnosis, inability to speak the local language well enough to complete the tests without an interpreter, active drug abuse, and wheel-chair bound. The same exclusion criteria apply for the MI-controls with the addition of having suffered a CA.

### Study procedure

All survivors in the main TTM2-trial are invited to a structured face-to-face follow-up visit 6 months after their OHCA where information on health-related quality of life, overall functional outcome, return-to-work, general physical function, global cognition, mental processing speed/attention and cardiovascular risk collected ^30^. As part of the cardiovascular risk assessment, OHCA-survivors answer two self-reported questions about their physical activities (Table 1) based on recommended levels for primary 4, 7 and secondary prevention of CAD 5, 6, 7, 8, 9, 10, 11, 12. At the end of the main trial follow-up, participants at selected TTM2-sites are provided written and verbal information about this sub-study (clinicatrials.gov NCT03543332) and are invited to participate.

**Table 1:** Two self-reported questions on physical activity.

	Questions on physical activity	Examples given to the participants
**Question 1** Physical activity	In the last week, how many days have you engaged in moderate physical activity for at least 30 min a day? (could be performed in blocks that last for at least 10 min adding up to a total of 30 min or more)	Moderate activities denotes activities performed for at least 10 min that leads to a moderate level of effort and a noticeably accelerate of heart rate. Examples of activities on this level includes a brisk walk, heavy cleaning, washing windows, cleaning the car, carpentry, bicycling with light effort, golf, swimming leisurely or similar. For more examples, see Haskell et al. 2007. Circulation ^4^.
**Question 2** Physical exercise training	In the last week, how many days have you engaged in vigorous (intense) intensity aerobic physical activities for at least 20 min (in one block)?	Vigorous-intensity aerobic physical activities are an activity that leads to a substantial increase in heart rate and e.g. causes rapid breathing. Examples of activities at this level includes jogging, running, walking very brisk, shoveling/digging, bicycling with a moderate effort/fast, swimming moderate/hard, tennis or similar. For more examples, see Haskell et al. 2007. Circulation ^4^.

For those who consent, an additional follow-up meeting is scheduled within approximately 4 weeks, i.e. 6−7 months after the CA. Before this meeting, questionnaires regarding anxiety symptoms, depression symptoms, kinesiophobia and fatigue are distributed to the participant by regular mail. At the meeting, participants return the questionnaires and are provided an accelerometer to wear for one week as an objective measure of physical activity. During the same week, participants are asked to register their physical exercise training in a diary. When the entire accelerometer-registration is completed, they answer the two self-reported questions about physical activity once again and return them in a pre-paid envelope together with the accelerometer and the training diary (Table 2).

**Table 2:** Overview of the different outcome measures for the TTM2 main trial 6 months after out-of-hospital cardiac arrest (OHCA) and for the sub-study 7 months after OHCA and myocardial infarction (MI).

		OHCA		MI-controls
Area	Instrument	TTM2 main trial after 6 months	Sub-study after 7 months	Sub-study after 7 months
Self-reported physical activity	Two self-reported questions	X	X	X
General physical function	Timed Stands Test (TST)	X		X
Global cognition screening	Montreal Cognitive Assessment (MoCA)	X		X
Mental processing speed/attention	Symbol Digit Modalities Test (SDMT)	X		X
Self-reported anxiety and depression symptoms	Hospital Anxiety and Depression Scale (HADS)		X	X
Self-reported kinesiophobia	Tampa Scale for Kinesiophobia Heart (TSK-Heart)		X	X
Self-reported fatigue Five dimensions: general fatigue, physical fatigue, mental fatigue, reduced activity, reduced motivation	Multidimensional Fatigue Inventory (MFI-20)		X	X
Objective physical activity	Accelerometer Actigraph		X	
Physical exercise training with rating of perceived exertion (RPE)	Training diary of physical exercise used together with the Borg Rating of Perceived Exertion Scale		X	

MI-controls are recruited from a cohort of participants with confirmed and treated acute ST-elevation or Non-ST-elevation MI who received coronary angiography, but without occurrence of CA. They are matched according to the following criteria in descending order: 1) country 2) date of OHCA/MI 3) gender and 4) age. If a MI-control participant declines the second-best match is approached to obtain a 1:1 ratio between OHCA-survivors and MI-controls. MI-controls are recruited from one site in each country.

The MI-controls are invited to a single face-to-face visit approximately 6−7 months after their MI and receive questionnaires regarding anxiety symptoms, depression symptoms, kinesiophobia and fatigue two weeks before this meeting by regular mail. At the follow-up visit, they return the questionnaires and perform the same tests on general physical function, global cognition and mental processing speed/attention as the OHCA-survivors complete in the main TTM2 trial follow-up (Table 2). Information about cardiovascular risk factors, including the two self-reported questions regarding physical activity are also collected.

The first participant of this study was included in July 2018. So far, 105 OHCA-survivors and 88 MI-controls are included. This study is coordinated with another TTM2 sub-study investigating neuropsychological function (clinicaltrials.gov NCT03543371) ^31^.

### Outcome measures

An overview of the different outcome measures is provided in Table 2. The main difference between OHCA-survivors and MI-controls is that the MI-controls are only seen once at 7 months after the MI. OHCA-survivors will complete the measures both at the main TTM2 follow-up, at the sub-study meeting, during and after which they wear the accelerometer (see Table 2). MI-controls do not wear the accelerometer or complete the training diary.

#### Self-report physical activity

The two self-reported questions on physical activity used in the main TTM2 follow-up (Table 1) will allow for categorization of the OHCA-survivors into three ordered groups of physical activity: physical activity below recommended levels for primary prevention, physical activity to a level recommended for primary prevention, or physical activity to a level recommended for primary prevention and secondary prevention for CAD 4, 5, 6, 7, 8, 9, 10, 11, 12 (Table 3). The self-reported questions were designed for the TTM2-trial and their psychometric properties have not been tested.

**Table 3:** Categorization on physical activity through self-reported questions and accelerometer.

Self-reported questions		Accelerometer Actigraph
Physical activity below recommended levels for primary prevention	<5 on question 1 OR <3 on question 2	≤150 min of moderate intensity or 75 min of vigorous intensity physical activity
Physical activity to a level recommended for primary prevention	≥ 5 on question 1 OR ≥ 3 on question 2	≥ 150 min of moderate intensity or 75 min of vigorous intensity physical activity in at least 10 min bouts
Physical activity to a level recommended for primary, and secondary prevention for CAD	≥2 on question 1 AND ≥3 on question 2	≥ 150 min of moderate intensity or 75 min of vigorous intensity physical activity in at least three 20 min bouts

150 activity minutes = 150 min of moderate intensity or 75 min of vigorous intensity physical activity or an equivalent combination thereof a week ^7^. Coronary artery disease (CAD).

#### General physical function

The Timed Stands Test (TST) is an objective measure of lower extremity strength, used as a rapid measure of general physical function 32, [33]. The participant is asked to rise 10 times to straight upright position from sitting, as quickly as possible, while the time is measured to the nearest 10th of a second. A shorter time indicates a better functioning of the lower extremity muscles. Age and gender adjusted norm values are available ^32^. The test will be interrupted after 60 s to avoid participants’ exhaustion and maximum time assigned to participants unable to complete 10 stands. Participants will be categorized into three groups according to performance: 1) normal level ^32^, 2) lower than expected given the age- and gender cut-offs but within 60 s and 3) do not manage within 60 s or at all. TST has been validated for rheumatoid arteritis and other chronic diseases ^33^ but not for CAD-patients or OHCA-survivors.

#### Global cognition

The Montreal Cognitive Assessment (MoCA) is used and recommended for cognitive screening after CA ^34^. The MoCA contains 11 sub-tasks of several cognitive domains as executive functioning, short-term memory and delayed recall combined into a total maximum score of 30. We use the full version 7.1–7.3 and add 1 point for participants who have 12 years or fewer of formal education according to the manual ^35^. A score ≥26 points is considered to be within the average range.

#### Mental processing speed/attention

Processing speed/attention will be assessed with the Symbol Digit Modalities Test (SDMT) ^36^. It was found to discriminate between OHCA-survivors and MI-controls in a previous study ^21^. The total amount of correct answers within 90 s are calculated and transformed into age and education adjusted z-scores. Scores ≤1.5 SD below the mean indicate impaired function ^37^.

#### Anxiety and depression symptoms

Information on emotional difficulties will be collected using the Hospital Anxiety and Depression Scale (HADS) ^38^. HADS is a self-report questionnaire validated in CAD-patients 39, 40 and previously administered to CA-survivors ^22^. It consists of 14 questions, 7 related to anxiety symptoms (HADS-A), and 7 related to depression symptoms (HADS-D). Each item is scored from 0 to 3 and a total sum score for each subscale (anxiety symptoms and depression symptoms) is calculated (range 0–21) with a cut-off of >7 indicating clinically significant difficulties ^38^.

#### Kinesiophobia

The Tampa Scale for Kinesiophobia Heart (TSK Heart) will be used to evaluate self-reported kinesiophobia. TSK Heart is a modified version of the original Tampa Scale ^41^ aimed at detecting kinesiophobia in patients with CAD. The questionnaire includes 17 statements/questions added into a total score that range from 17 to 68. Values >37 represent a high level of self-reported kinesiophobia ^17^. TSK Heart has adequate reliability and validity for patients with CAD ^42^.

#### Fatigue

The Multidimensional Fatigue Inventory (MFI-20) is a 20-item self-reported questionnaire that measures fatigue over the last few days in five dimensions: general fatigue, physical fatigue, mental fatigue, reduced activity and reduced motivation. Sum-scores are calculated for each dimension separately and range from 4 to 20, higher scores indicates a higher level of fatigue ^43^. MFI-20 has previously been used in a sample of MI-patients ^18^, but not for patients with OHCA.

#### Physical activity, objective assessment

The OHCA-survivors physical activity will be objectively evaluated by the accelerometer Actigraph wGT3X-BT (Actigraph, Florida, USA), found to be an instrument with good validity to assess physical activity among persons with chronic diseases including CAD ^44^. The Actigraph wGT3X-BT measures wear time, steps and intensity of physical activity. This data can be used to calculate the duration and frequency of physical activity in different intensities. The OHCA-survivors will wear the Actigraph for 7 consecutive days. The analysis will include all participants with a minimum of 4 valid days of assessment ^45^. A valid day will be defined as a minimum of 10 h wear time. The Actigraph is programmed to start at 7.00 AM on the day after the sub-study follow-up meeting and stops exactly 7 days later. The accelerometer will be placed over the right hip with an elastic belt and removed when the participants are sleeping or in contact with water 44, 45, 46, 47, 48. The participants will receive verbal and written information, including information on how to contact study team if necessary during this week, to minimize the risk of missing data.

To analyze the information collected by the accelerometer, the ActiLife software version 6.16.3 will be used with the following settings: normal filter, 30 Hz sampling frequency and 60 s epoch length ^45^. As there is currently no specific accelerometer cut points for OHCA-survivors, the commonly used accelerometer cut points from Freedson Adult ^49^ will be used to analyze minutes of physical activity in moderate and vigorous intensity. The results will be used to categorize the OHCA-survivors into three ordered groups of physical activity (Table 3).

#### Training diary of physical exercise training

Participants will be asked to fill in a pre-printed training diary of physical exercise, structured as an activity log, including activities they consider as physical exercise training. This is defined as physical activity that is planned, structured and repetitive and aims to improve or maintain physical fitness ^50^. Information about what constitutes physical activity and exercise is given to the participant verbally during the follow-up appointment and explained in writing in the training diary. The aim of the training diary is to add information on activities that cannot be measured by the accelerometer e.g. swimming. Participants will specify the type of activity, duration and rate the level of exertion according to the Borg Rating of Perceived Exertion Scale (RPE). The RPE measures exertion on a scale from 6 to 20, where 6 denotes "no exertion at all" and 20 denotes "maximal exertion”^51^.

#### Socio-demographical and medical characteristics for the participants

Socio-demographic and medical characteristics for the OHCA-survivors will be obtained from the TTM2 database. The same information will be obtained from the MI-controls at the follow-up meeting (Table 4).

**Table 4:** Clinical and medical participants’ characteristics that will be presented for the OHCA-survivors and MI-controls.

Sociodemographic background	
Age	Years
Gender	Male, female
Level of education	No formal education, incomplete primary/lower secondary school, complete primary/lower secondary school, incomplete upper secondary school, complete upper secondary school, some university-level education without degree, university-level education with degree
Living situation	Home, hospital, rehabilitation center, nursing home, other
Working status before OHCA/MI	Working full-time, or part-time, unemployed, retired due to age, or due to disabilities/health problems, on sick-leave, other
**During hospitalization**	
LVEF	Percent
ICU length of stay	Days
Days at hospital	Days
**At 6−7 months follow-up**	
Working status	Working full-time, or part-time, unemployed, retired due to age, or due to disabilities/health problems, on sick-leave, other
Current smoking	no/yes
Current diabetes	no/yes
Total cholesterol	mmol/L
HDL cholesterol	mmol/L
Systolic blood pressure	mmHg
HbA1c	mmol/mol
BMI	pounds/inches^2^, kg/cm^2^
Any current pharmaceutical treatment for hypercholesterolemia	no/yes
Any current pharmaceutical treatment for diabetes	no/yes
Any current pharmaceutical treatment for high blood pressure	no/yes
General physical function	Timed Stands Test (TST)
Global cognition (screening)	Montreal Cognitive Assessment (MoCA)
Mental processing speed/attention	Symbol Digit Modalities Test (SDMT)
Self-reported anxiety and depression symptoms	Hospital Anxiety and Depression Scale (HADS)
Self-reported kinesiophobia	Tampa Scale for Kinesiophobia Heart (TSK-Heart)
Self-reported fatigue	Multidimensional Fatigue Inventory (MFI-20)

Out-of-hospital cardiac arrest (OHCA), myocardial infarction (MI), left ventricular ejection fraction (LVEF), high-density lipoprotein (HDL), hemoglobin A1c (HbA1c), body mass index (BMI).

#### Ethics

The TTM2-trial (EPN-reference number 2015/228) ^28^ and this sub-study (EPN-reference number 2017/933) was approved by the Regional Ethics Committee at Lund University, Sweden and corresponding ethics committees in the United Kingdom and Denmark. Written informed consent to participate in the sub-study is obtained before the sub-study follow-up for both OHCA-survivors and MI-controls.

### Statistical analysis plan

Data will be presented according to the STROBE-guidelines. Descriptive information and patient characteristics for OHCA-survivors and MI-controls (Table 4) will be presented, with potential differences between the two groups described. Information on missing data will be provided.

#### Analyses for hypothesis 1

The OHCA-survivors’ self-reported physical activity, obtained at the main TTM2-trial follow-up, will be compared to the self-reported physical activity of the MI-controls, obtained at the sub-study meeting. The three ordered categories of physical activity will be used in the analyses (Table 3). Numbers and percentages of participants in each category will be presented and potential differences in level of physical activity between OHCA-survivors and MI-controls will be analyzed using the non-parametric Mann Whitney *U* test.

#### Analyses for hypothesis 2

Physical activity will for these analyses be dichotomized into two categories; not fulfilling the primary prevention or fulfilling at least primary prevention recommendations. Single and multivariable logistic regressions will be used to identify predictors of not fulfilling the primary prevention recommendations. Pre-specified potential predictors are: older age, female gender, problems with general physical function (TST), global cognition (MoCA), mental processing speed/attention (SDMT z-scores), anxiety symptoms (HADS-A), depression symptoms (HADS-D), kinesiophobia (TSK-heart) and fatigue (MFI-20). Effect sizes will be reported as odds ratios.

#### Analyses for hypothesis 3

In the OHCA-group, retrospective self-reported levels of physical activity will be compared to objective measurements obtained during the same week as recorded by the accelerometer. Both datasets will be categorized as described in Table 3 to allow cross tabulation and analysis. Percent agreement and Cohen’s kappa will be calculated. At least moderate agreement is by Cohen’s kappa defined as 0.41−0.60 “moderate”, 0.61−0.75 “substantial” and 0.80–1.00 “almost perfect” 52, 53.

#### Sample size calculation

The Swedish national registry of MI-patients ^54^ use identical two self-reported questions on physical activity as in this study. In our local registry data, 20% of the MI-patients were below recommended levels for primary prevention of physical activity, 52% were at a physical activity level recommended for primary prevention and 28% were physically active to a level recommended for both primary and secondary prevention of CAD.

In lack of published data on physical activity for OHCA-survivors, we used unpublished data on self-reported physical activity (SF-36v2, Physical Function-subscale) from a previous trial ^21^. This led to an assumption that 30% of the OHCA-survivors were below recommended levels for primary prevention and that 12% were physically active to a level recommended for primary and secondary prevention of CAD.

Using these two assumed distributions of ordered data and a Mann-Whitney U test, 105 participants are required in each group for a statistical power >80% to detect differences in physical activity between the OHCA survivors and MI-controls at p = 0.05. Assuming a loss of 5%, we aim to include 110 participants in each group.

Due to the explorative design of this study, p-values <0.05 will be used to indicate potentially important differences.

## Discussion

We describe a sub-study to a large clinical trial in which physical activity is assessed by two self-reported questions as a part of the OHCA-survivors’ cardiovascular risk. This sub-study was designed to complement the main trial by the addition of an objective measure of physical activity and a control group with similar level of CAD, but without the problems related to hypoxic-ischemic brain injury and/or the post-intensive care stay. To our knowledge, this is the first multicenter study specifically focusing on physical activity after OHCA.

The current recommendations for physical activity after CAD are based on self-reports and consensus decisions 3, 4, 5, 6, 7, 8, 9, 10, 11, 12, but information about how well self-reported questions correspond to the actual performed physical activity among OHCA-survivors is unknown. Modern techniques have enabled quantification of physical activity in an objective way.

Little comparable data exist for accelerometer data for the cardiac population ^55^, none for OHCA. Accelerometer cut points for different intensities of physical activity may differ between patient populations, influenced by age and exercise capacity. We have chosen the most used accelerometer cut points available ^49^ but we recognize that they are based on young healthy adults, and were found to overestimate sedentary-light intensity physical activity and underestimate moderate-vigorous intensity physical activity in older adults ^56^. The accelerometer does not measure the intensity of all activities for example resistance training. To add some descriptive information to lessen this limitation participants are encouraged to report all their physical exercise training in the training diary.

Our matching criteria for the MI-controls were developed to enable recruitment of participants with similar degree of CAD and pre-event related risk-factors as our OHCA-survivors since this is the group for whom current cardiac rehabilitation programs have been designed. We do not know if current cardiac rehabilitation programs are appropriate for OHCA-survivors. Admittedly, we lack information on the participants’ physical and cognitive functional status before the OHCA/MI. We aim to present descriptive information regarding the two groups as reported in Table 4.

## Conclusion

This study will provide important information on the levels of physical activity after OHCA in relation to recommended levels for primary and secondary prevention of CAD, and explore predictors of importance for physical inactivity. In addition, our results will provide information on how to measure physical activity in OHCA-survivors.

## Credit author statement

All authors have made substantial contributions to the manuscript. KH, GL, ÅBT, TC and NN designed the sub-study. KH is the principal investigator. KH, TC, GL, ÅT, SU, EBN, NN and HF are the coordinating team. CR, HK and MW are national investigators. LGØ, AMG, TRK, MM, MS and HH are site investigators. KH, EBN, MS, MM and HH conducted the follow-up meetings. SU is the trial statistician. KH, GL, ÅBT and TC drafted the manuscript. All authors revised and gave final approval to the manuscript.

## Sources of funding

This work is supported by grants from The Skane University Hospital Foundations 2019-o000032, 2019; The Skane University Hospital Vårdakademin 2018; Hans-Gabriel and Alice Trolle-Wachtmeister Foundation for Medical Research 2019 and the Gyllenstierna-Krapperup Foundation 2016−0051, 2016. The study sponsors have no involvement in the study design, in the collection of data, or in the forthcoming analyses and interpretation of data, writings of manuscript of manuscripts or in the decisions to submit manuscripts for publication.

## Conflicts of interest

KH, GL, ÅBT, SU, EBN, HF, NN, LGØ, AG, HH, TK, HK, MM, CR, MS, JU, MW, TC report conflicts of interest: None
